# Development of a Self-Assembled Hydrogels Based on Carboxymethyl Chitosan and Oxidized Hyaluronic Acid Containing Tanshinone Extract Nanocrystals for Enhanced Dissolution and Acne Treatment

**DOI:** 10.3390/ph15121534

**Published:** 2022-12-09

**Authors:** Xiaohan Tang, Yan Liu, Hailong Yuan, Rong Gao

**Affiliations:** 1 Department of Pharmacy, Air Force Medical Center, PLA, Beijing 100142, China; 2 School of Pharmacy, Anhui Medical University, Hefei 230032, China

**Keywords:** nanocrystals, self-assembled, hydrogels, acne, poorly water-soluble drug

## Abstract

This study aimed to construct a pH-responsive nanocrystalline hydrogel drug delivery system for topical delivery of insoluble drugs based on the self-assembly behavior of carboxymethyl chitosan (CMC) and oxidized hyaluronic acid (OHA). The tanshinone nanocrystal (TNCs) extract was prepared by dielectric milling method, the type and ratio of stabilizer of the drug were investigated to optimize the prescription, and the effector surface method was used to optimize the preparation process. OHA was prepared by the sodium periodate oxidation method, and the concentration of CMC and OHA was optimized using gel formation time as an indicator. OHA was dissolved in TNCs and self-assembled with CMC solution to form tanshinone extract nanocrystal hydrogels (CMC-OHA/TNCs), of which the physicochemical properties and in vitro antibacterial activity were evaluated. Results showed that the optimized prescription and process could produce tanshinone extract nanocrystals with a particle size of (223.67 ± 4.03) nm and a polydispersity index (PDI) of 0.2173 ± 0.0008. According to SEM and XRD results, TNCs were completely wrapped in the hydrogel as nanoparticles, and the crystallinity of TNCs was reduced and the diffraction peaks in CMC-OHA/TNCs almost disappeared. In vitro, transdermal test results showed that CMC-OHA/TNCs could release the drug continuously at the acne lesions. The cell-counting kit-8 (CCK-8) assay confirmed that the CMC-OHA/TNCs had no obvious cytotoxicity. The minimum inhibitory concentration (MIC) and minimum bactericidal concentration (MBC) of CMC-OHA/TNCs against *Propionibacterium acnes* and *Staphylococcus aureus* were significantly lower and the diameter of the inhibition circle was obviously higher than that of TNCs and tanshinone extract crude suspension. This study demonstrated that CMC-OHA/TNCs was a promising delivery system for topical delivery of insoluble drugs, which could improve the solubility of tanshinone extract and enhance its in vitro bacterial inhibitory activity.

## 1. Introduction

Acne is a widespread skin disease especially among teenagers with rates up to 85%, including 30% moderate and 10% severe acne [1]. Teenagers with acne experience many negative side effects, including discomfort, emotional stress, disfigurement, and permanent scarring. Additionally, it might cause patients anxiety, embarrassment, and can affect their physiological and social wellbeing [2]. The pathogenesis of acne is complex, and it was thought that the development of acne is mainly related to androgenic sebum production, altered sebum composition, abnormal keratinization of the follicular sebaceous ducts, colonization by *Propionibacterium acnes* (*P. acnes*) and *Staphylococcus aureus* (*S. aureus*), inflammatory response and immunity [3]. Recent studies had shown that genetic susceptibility and diet are also important factors in the development of acne [4,5,6]. Antibiotics were the first line of treatment for acne, but long-term use of antibiotics could induce the development of drug-resistant *P. acnes*, which is an important cause of acne flare-ups [7]. With the emergence of antibiotic resistance and the urgent need to develop new drugs, researchers were gradually focusing on the use of Chinese herbal medicine and had found that a variety of herbal extracts had good therapeutic effects on acne [8].

Tanshinone extract (TE), lipophilic active constituents extracted from *Salvia miltiorrhiza* Bge., with cryptotanshinone and tanshinone IIA as the main components, was considered to be one of the most promising drugs for the treatment of acne [9]. TE could treat acne in a variety of ways, including inhibition of dihydrotestosterone (DHT) synthesis, inhibition of the growth and reproduction of *P. acnes*, anti-inflammatory effects, inhibition of the production of inflammatory factors, improvement of microcirculation, increase in local microvascular perfusion, and promotion of the healing of the affected area [10,11]. However, TE had poor aqueous solubility which limited its therapeutic efficacy and clinical application.

Nanocrystals as a new delivery system of drugs with poor water solubility were one of the most successful nanotechnologies of the last 20 years in pharma [12]. The smaller size and larger surface area of nanocrystals lead to higher dissolution rate, hence increasing the oral bioavailability and absorption of poorly water-soluble drugs [13,14]. Nanocrystals also showed great potential in the field of local drug delivery. On the one hand, nanocrystals had a high drug loading capacity because they were made up of pure drug particles and required a low concentration of surfactants for stabilization. On the other hand, there was no requirement for the use of co-solvents or extreme pH range for solubilization [15]. Moreover, the increased dissolution rate and saturation solubility intensifies the concentration gradient between the biological membranes including stratum corneum and topical preparation, subsequently allowing higher penetration into skin layers [16]. Particularly, an increase in the particle curvature with a crystal size below 500 nm facilitates the penetration of intact drug particles into the skin through the hair follicles and its subsequent absorption by the surrounding follicular epithelium [17]. As a result, preparing TE nanocrystals (TNCs) seemed to be an ideal approach to improve its solubility.

Due to the weak skin retention at the site of infection of nanocrystals, loading them into a suitable carrier was essential for the treatment of acne. Some researchers had loaded silver sulfadiazine nanocrystals into a temperature-sensitive hydrogels of poloxamer 407 for the treatment of burns. The hydrogels had good biocompatibility and antibacterial properties [18]. Among the matrix materials for hydrogels, hyaluronic acid (HA) was a promising and excellent material. HA was a widely distributed component of the extracellular matrix of human tissues and had good biocompatibility and biodegradability [19]. Oxidized hyaluronic acid (OHA) was a product of the oxidation of HA. The active aldehyde group in OHA could self-assemble with the amino group of carboxymethyl chitosan (CMC) to form hydrogels (CMC-OHA hydrogels). The self-assembled hydrogels had excellent antibacterial properties, biodegradability, and non-toxicity [20]. In addition, the self-assembled hydrogels could break the Schiff base bond in a slightly acidic environment to release the drug and had a certain acid responsiveness, which was suitable for the slightly acidic environment of human skin. The self-assembled hydrogels had broad application prospects in the fields of medicine, food, packaging and environmental protection [21,22]. Accordingly, this pH-responsive CMC-OHA hydrogel was considered suitable as a carrier for local delivery of nanocrystals in this study.

Herein, the aim of this study was to formulate a CMC-OHA hydrogels containing TNCs (CMC-OHA/TNCs) for transdermal delivery of TE and enhancing its acne treatment effect. TNCs was prepared by the media milling method and optimized by single-factor experiment and Box–Behnken experimental design method. OHA was prepared by sodium periodate oxidation and then dissolved in nanocrystals and self-assembled with CMC solution to form CMC-OHA/TNCs. Physicochemical characterizations of CMC-OHA/TNCs were analyzed using X-ray diffraction (XRD) and scanning electron microscopy (SEM). In vitro transdermal assays were used to evaluate the ability of CMC-OHA/TNCs to release drugs at the acne site. Finally, the antibacterial activity and toxicity of CMC-OHA/TNCs were evaluated.

## 2. Results and Discussions

### 2.1. Optimization of TNCs Prescriptions

**Figure 1:**
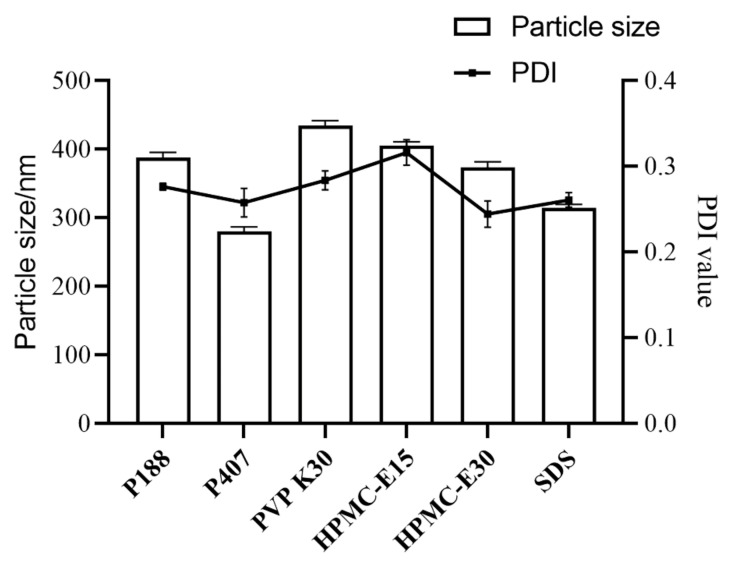
The effects of different stabilizers on the particle size and PDI of TNCs.

**Figure 2:**
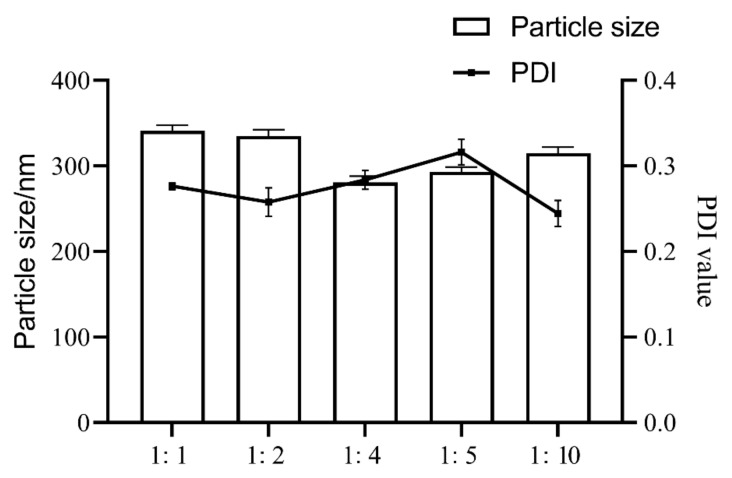
The effects of different ratios of P407 to TE on particle size and PDI values of TNCs.

We determined the particle size and polydispersity index (PDI) of the TNCs prepared with the six stabilizers, and the TNCs prepared with Poloxamer 407 (P407) showed a smaller particle size and a suitable PDI (Figure 1). P407, a nonionic surfactant, was commonly used in topical formulations because it was less irritating compared with ionic surfactants. The effect of the amount of stabilizer was also investigated; the results showed that insufficient stabilizer promoted agglomeration or aggregation, while using too much stabilizer could cause Ostwald ripening. In addition, drug dosage, type and the ratio of the stabilizer could change the pharmaceutical properties of the system; for instance, viscosity could affect the efficiency of nanosuspension production. Figure 2 shows that when the ratio of P407 to TE decreases from 1:1 to 1:10, the particle size first decreases and then increases, and taking particle size and PDI considered, a ratio of 1:4 between P407 and TE was chosen as the best prescription. The short-term stability of the TNCs was investigated by monitoring particle size and PDI at 25 °C. The measurements of the particle size and PDI of TNCs were performed initially and at one day, one week, one month, and three months during the storage. The results showed that the TNCs maintained a relatively stable particle size and PDI over a period of three months, which demonstrated that using P407 as the stabilizer could completely cover the TE surface and provide sufficient spatial repulsion between the particles for optimum stability of the nanosuspension.

### 2.2. Optimization of TNC Processes

A total of 17 experiments in Box–Behnken design were required, and the independent variables (the amount of grinding media, grinding speed and grinding time) and responses of the 17 runs are provided in Table 1. The regression analysis was performed using Design Expert software; as a result, the particle size ranged from 220.8 to 338.0 nm and PDI ranged from 0.2148 to 0.2616. For Y_1_, R^2^ = 0.9776, adjusted R^2^ = 0.9489, predicted R^2^ = 0.8991, C.V.% = 2.72. For Y_2_, R^2^ = 0.9727, adjusted R^2^ = 0.9375, predicted R^2^ = 0.8266, C.V.% = 1.60. The variables with *p* < 0.05 were involved in the equation of Y_1_ and Y_2_:

Y_1_ = 264.936 − 29.835 X_1_ + 6.336 X_2_ + 15.057 X_3_ − 25.578 X_2_X_3_ − 14.022 X_1_^2^ + 31.242 X_3_^2^; Y_2_ = 0.21792 + 0.00435 X_2_ − 0.0063 X_1_X_2_ − 0.0102 X_1_X_3_ − 0.0168 X_2_X_3_ + 0.00459 X_2_^2^ + 0.01749 X_3_^2^.

**Figure 3:**
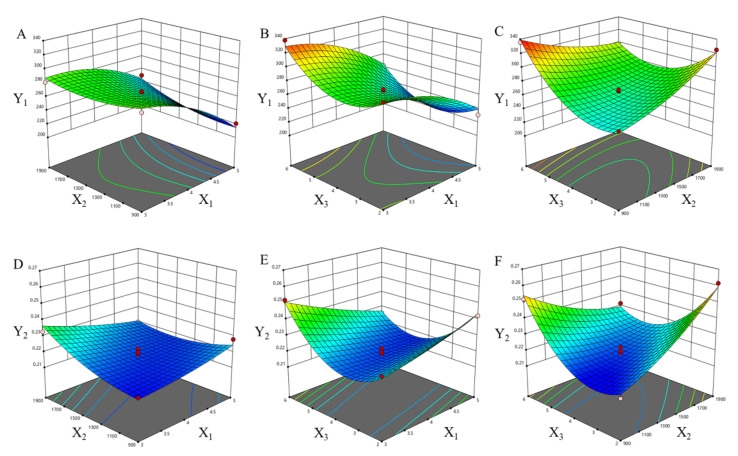
Three-dimensional contour plot showing the effect of independent variables on response of particle size and PDI of TNCs. (**A**) X1 and X2 on response Y1, (**B**) X1 and X3 on response Y1, (**C**) X2 and X3 on response Y1, (**D**) X1 and X2 on response Y2, (**E**) X1 and X3 on response Y2, (**F**) X2 and X3 on response Y2. 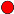, design points above predicted value; ○, design points below predicted value.

The type of interactions among the three tested variables and the relationship between responses and experimental levels of each variable could be illustrated in 3D response surface plots, shown in Figure 3. The amount of grinding media and grinding speed remarkably influenced the particle size (Table 2, Figure 3A–C). The results showed that the particle size decreased non-linearly with the increase in the amount of grinding media and the grinding speed, and also influenced by the grinding time. Moreover, the PDI decreased nonlinearly with the amount of grinding media, and decreased first and then increased with grinding speed and grinding time.

It could be seen that the effect of each factor on the size of the drug was not a simple linear relationship. With the increase in grinding speed and grinding time, the size of the pieces decreased and the size distribution became more uniform. The particle size dropped sharply from microns to approximately 200 nm when the grinding time was prolonged, but the size increased slightly when the grinding time was further prolonged, which may have been caused by the secondary agglomeration of the nanoparticles. The phenomenon occurred commonly during milling processes and other top-down nanosizing technologies. By mechanically breaking the microparticles at weak spots, the comminution process broke them in a more uniform manner. During the comminution process, there was a gradual decrease in imperfections, and the remaining crystals were becoming more and more perfect. When the mechanical force was equal to the interaction forces in the crystal, the particles were hard to ground further [23].

After analyzing the polynomial equations and depicting the dependent and independent variables, a further optimization and validation process by means of the Design Expert software was undertaken with desirable characteristics to probe the optimal process of TNCs. This depended on the prescriptive criteria of a minimum value of particle size and a minimum value of PDI. Using a Design Expert 8.0 software optimization process, the selected values of X_1_-X_3_ were 5 mL, 1360 rpm and 4.1 h, respectively, which produced theoretical values of 220.8 nm of particle size and 0.2177 of PDI, respectively. Therefore, in order to confirm the predicted model, a new batch of TNCs according to the optimal processes factor levels was prepared. Finally, three verification experiments were performed using the optimal process, and the particle size was (223.67 ± 4.03) nm and PDI was 0.2173 ± 0.0008, which was close to the predicted value (*p* > 0.05) indicating the reliability of BBD to predict the desirable nanocrystal processes.

### 2.3. Preparation and Characterization of CMC-OHA/TNCs

**Figure 4:**
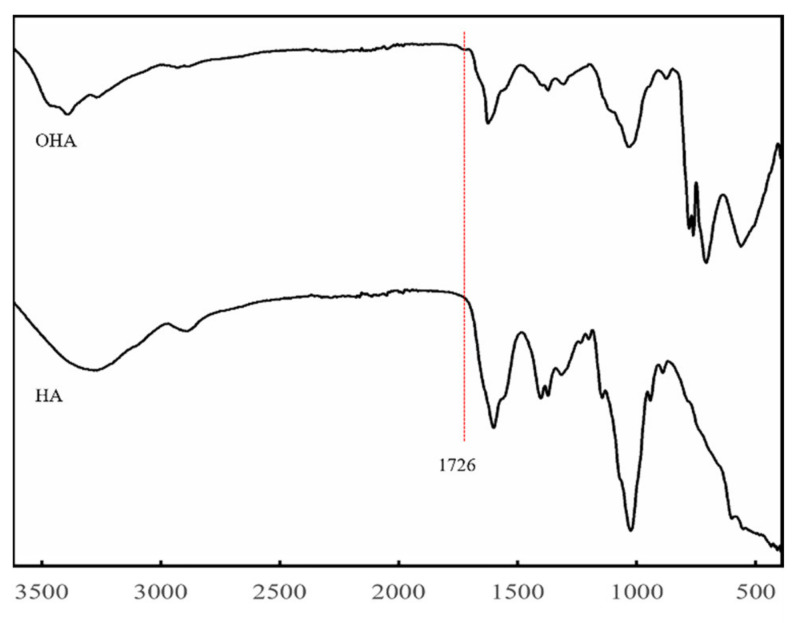
FT-IR spectra of HA and OHA.

**Figure 5:**
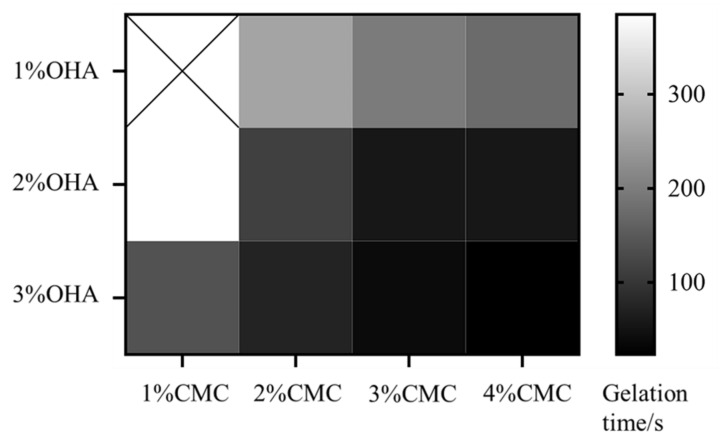
Gelation time of the hydrogels formed with different concentrations of CMC and OHA.

The oxidized HA was obtained by a ring-opening reaction using sodium periodate, which resulted in the introduction of the dialdehyde group into the HA dimer unit. As shown in Figure 4, there was a new peak at 1726 cm^−1^ in the Fourier transform infrared (FT-IR) spectra of OHA compared to the FT-IR spectra of HA. In the FT-IR spectrum of OHA, the peak at 1726 cm^−1^ was the stretching vibration of the -C=O- bond of the aldehyde group, which could prove the successful preparation of OHA [24]. The gel formation time of each group of hydrogels was shown in Figure 5. It could be found that the concentration of both OHA and CMC had a significant effect on the gel formation time, and the gel formation time gradually shortened with the increase in concentration of OHA and CMC. To form stable hydrogels, the concentration of OHA and CMC must be at least 1% (*w*/*v*). When the concentration of OHA was 1%, the gel formation time reduced from 261.67 s to 176.33 s as the concentration of CMC increased from 2% to 4%. When the concentration of OHA was 3%, the gel formation time was further reduced from 152.00 s to 24.00 s as the concentration of CMC was increased from 1% to 4%. Further increasing the concentration would increase the preparation time of the precursor solution, resulting in an increase in the overall preparation time. Therefore, 3% OHA and 4%CMC were ultimately preferred for subsequent studies.

The possible reason is that the reactive amino group in CMC and the reactive aldehyde group in OHA could self-assemble to form hydrogels by Schiff base reaction, so the gel formation time was mainly related to the number of reactive groups in the solution. When the concentration of both CMC and OHA was 1%, the number of reactive groups in the solution was too small to form a hydrogel, and increasing the concentration led to a greater number of reactive groups in the solution, which solidified quickly to form a stable hydrogel.

**Figure 6:**
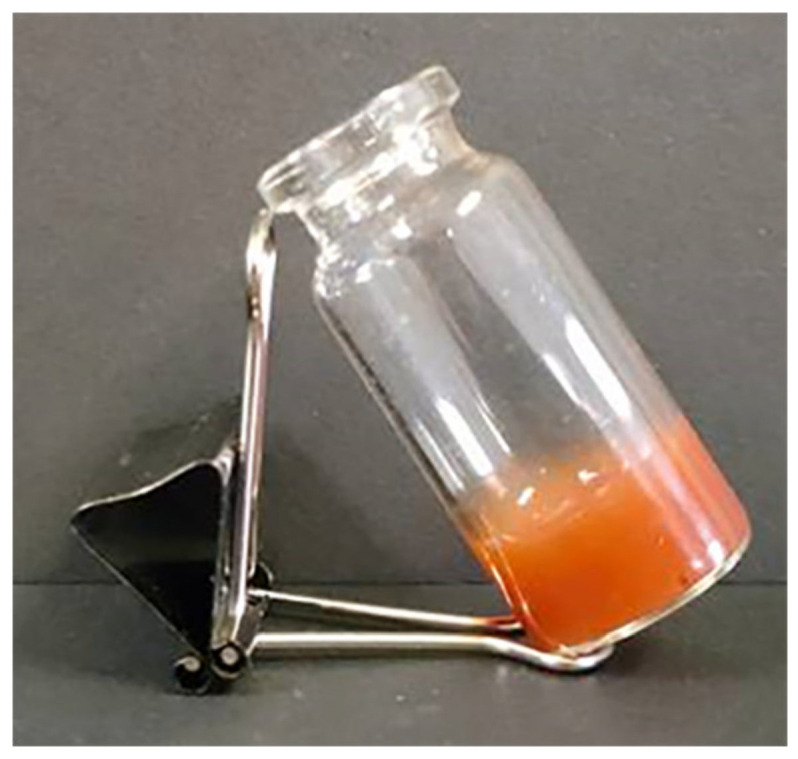
Appearance of CMC-OHA/TNCs.

CMC-OHA/TNCs prepared by optimal prescription showed homogeneous appearances and good gel formation (Figure 6). The gelling time of CMC-OHA/TNCs was (24.41 ± 0.91) s, which was not significantly different from the gelling time of the blank hydrogel matrix (*p* > 0.05).

### 2.4. Morphology of CMC-OHA/TNCs

**Figure 7:**
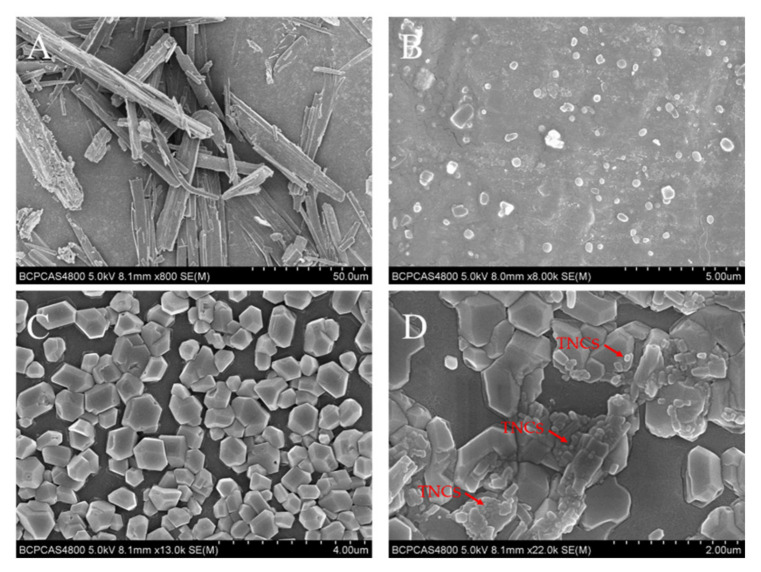
SEM photographs of TE powders (**A**), TNCs (**B**) CMC-OHA hydrogels (**C**) and CMC-OHA/TNCs (**D**).

SEM images of TE powders, TNCs, CMC-OHA hydrogels, and CMC-OHA/TNCs are shown in Figure 7. TE powder showed irregular shape with a great deal of angularities (Figure 7A). The particle size of the TNCs was significantly reduced compared to that of the TE powder, mostly in a round-like shape with some agglomeration (Figure 7B). The particle size of the TNCs was approximately 200 nm, which was consistent with the results of the Zeta-sizer assay. The microscopic images of the blank hydrogels and the CMC-OHA/TNCs after dried at room temperature showed that the TNCs were encapsulated in the hydrogels matrix, demonstrating that the TNCs remain in the hydrogels at the nanoscale size (Figure 7C,D).

It had been found that the addition of nanosuspension to gel matrices had led to the growth of crystals larger than 1 µm in diameter [25]. Therefore, loading TNCs into the hydrogels and maintaining the nanocrystal morphology was crucial to prepare CMC-OHA/TNCs. The self-assembly process of hydrogels did not alter the nanocrystal morphology, and although there were aggregations of nanocrystals in the hydrogels, most of them remained nanoscale in size. This may have been caused by mild reactions during in situ crosslinking between CMC and OHA, resulting in a cavity structure in the hydrogel where nanocrystal encapsulation is not disrupted.

### 2.5. X-ray Powder Diffraction (XRD) of CMC-OHA/TNCs

**Figure 8:**
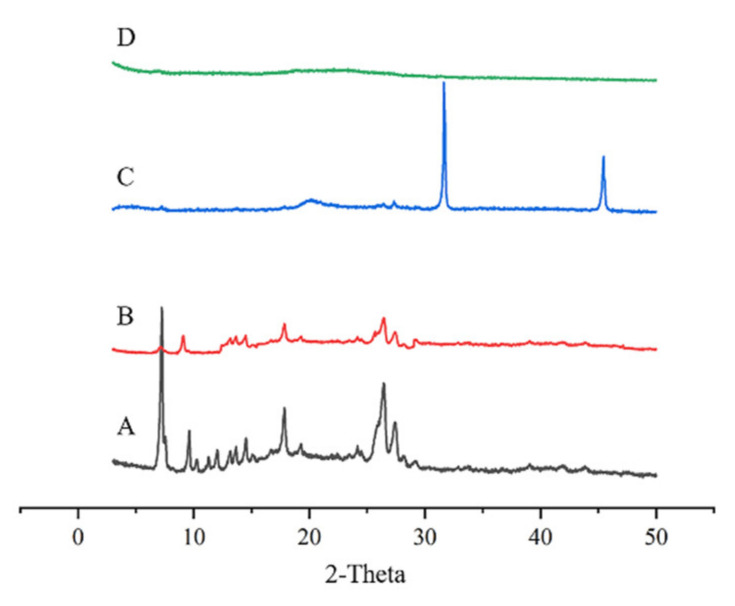
XRD for TE (**A**), TNCs (**B**), OHA and CMC powders (**C**) and CMC-OHA/TNCs (**D**).

XRD was performed to further analyse the crystalline nature of TE powder, TNC freeze-dried powder, and CMC-OHA/TNCs. The XRD pattern of TE powder showed characteristic peaks at locations such as 2θ value of 7.2°, 9.5° and 26.4°, while the peaks of TNCs at these locations were significantly weaker (Figure 8A). Figure 8C shows the XRD patterns of OHA and CMC powders. Two sharp peak shapes could be observed at 2θ 31.6° and 45.5°, while these two peaks completely disappear in Figure 8D, proving that self-assembly of CMC and OHA occurred to form CMC-OHA/TNCs. In addition, the peaks at 2θ 7.2° in Figure 8D weaken further, while the peaks at 2θ value of 9.5° and 26.4° disappear completely.

The XRD results showed that the XRD patterns of TNCs and TEs were essentially the same and no new crystal diffraction peaks appeared, indicating that the crystalline state of TE did not change during the media milling process. The intensity of crystallinity could be reflected by the intensity of the diffraction peaks. The vibrational intensity of the diffraction peaks of TNCs was significantly lower than that of TE. This phenomenon could be explained by a significant decrease in crystallinity due to a reduction in drug particle size. It was confirmed that drugs with low crystallinity and small particle sizes are easier to dissolve and absorb [26]. In addition, the further attenuation of CMC-OHA diffraction peaks demonstrated that the TNCs was successfully encapsulated by the CMC-OHA hydrogels.

### 2.6. In Vitro Transdermal Assays

**Figure 9:**
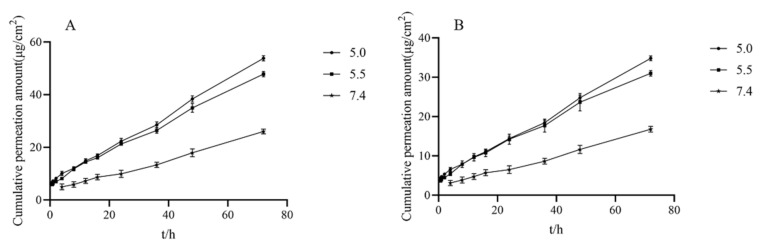
In vitro transdermal of tanshinone IIA (**A**) and cryptotanshinone (**B**) at different pH receptive solutions (pH = 5.0, 5.5, and 7.4).

To understand the beneficial effect of the CMC-OHA/TNCs in delivering TE, we investigated the in vitro skin permeation of TE released by CMC-OHA/TNCs. The cumulative permeation of CMC-OHA/TNCs was significantly higher at pH 5.0 and 5.5 than at pH 7.4 (Figure 9). The cumulative release of tanshinone IIA was (53.82 ± 0.77) and (47.82 ± 0.83) μg/cm^2^ at pH 5.0 and 5.5, respectively, which was much higher than that of (26.04 ± 0.77) μg/cm^2^ at pH 7.4 (Figure 9A). The cumulative permeation of cryptotanshinone from the receiver cell at pH 5.0 and 5.5 were (34.83 ± 0.50) and (31.01 ± 0.57) μg/cm^2^, respectively, which was higher than that of (16.77 ± 0.61) μg/cm^2^ at pH 7.4 (Figure 9B).

The acid responsiveness of CMC-OHA hydrogels was derived from the Schiff base imine bond in their structure. The rapid and reversible pH responsiveness of the hydrogel was due to the hydrolysis of the Schiff base under acidic conditions. The hydrogel exhibited excellent stability in neutral environments, but a gel-to-solution transition occurred as the ambient pH shifted to weakly acidic. Furthermore, in a weakly acidic environment (pH 6.0–7.4), a slight pH change (0.2) could lead to significant changes in the mechanical, dissolution and drug release properties of the hydrogel [27]. Some researchers had suggested a slight increase in skin pH at the acne site, but this study was inconclusive due to the inconsistency of skin pH measurement methods and the dynamics of human facial pH. Skin pH was found to remain between 5.04 and 5.52 before and after treatment at the acne site [28]. Therefore, CMC-OHA/TNCs was examined in vitro transdermal experiments at pH conditions of 5.0, 5.5 and 7.4. Clearly, CMC-OHA/TNCs could release drugs responsively at the site of acne lesions.

### 2.7. In Vitro Cytotoxicity of CMC-OHA/TNCs

**Figure 10:**
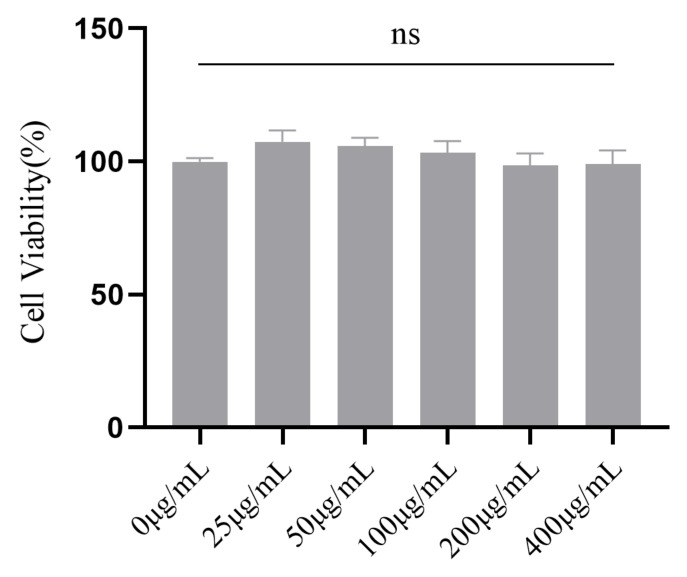
Cell viability of 3T3-L1 following incubation with CMC-OHA/TNCs for 48 h. ns = non-significant.

The favorable biocompatibility of a hydrogel is a prerequisite for biomedical applications. Accordingly, the cytotoxicity of CMC-OHA/TNCs was investigated using a cell counting kit-8 (CCK-8) assay [29]. As expected, there was no cell toxicity under different concentrations of CMC-OHA/TNCs (25–400 μg/mL) after 48 h of incubation (Figure 10). The mean fold changes were 107.47%, 105.82%, 103.31%, 98.53% and 99.02% for the concentrations at 25–400 μg/mL, respectively, which were not statistically significant compared to the control (*p* > 0.05).

### 2.8. In Vitro Antibacterial Activity Assay

The minimum inhibitory concentration (MIC) and minimum bactericidal concentration (MBC) values of TE crude suspension and TNCs against *P. acnes* and *S. aureus* were further detected, and the results showed that the antibacterial activity of the TNCs was significantly increased compared to the TE crude suspension (Table 3). In addition, the MIC and MBC of CMC-OHA/TNCs against *P. acnes* and *S. aureus* were further reduced compared to TE crude suspension and TNCs. The MIC values of TE crude suspension were 125 μg/mL against *P. acnes* and *S. aureus*, while they were 31 and 63 μg/mL for TNCs and 16 and 31 μg/mL for CMC-OHA/TNCs. The MBC values of the crude suspension of TE were 250 and 64 μg/mL for *P. acnes* and *S. aureus*, while they were 63 μg/mL for the TNCs and 63 and 31 μg/mL for CMC-OHA/TNCs. The improved antimicrobial effect of the nanoparticles probably occured due to their small size and high surface area ratio, which enabled them to interact closely with the microbial film [30].

**Figure 11:**
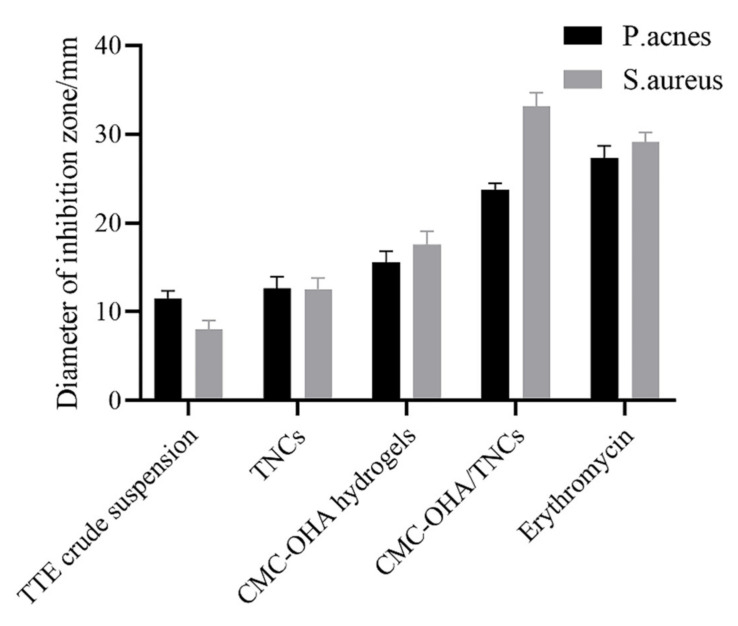
Antibacterial activity of CMC-OHA/TNCs in vitro.

To better compare the bactericidal effect of TNCs and TE crude suspension, we further measured the values of the zone of inhibition, and the results revealed that the bactericidal effect of TNCs was significantly increased compared with that of TE crude suspension against both selected bacteria (Figure 11). The TE crude suspension had an inhibition circle diameter of (11.54 ± 0.74) mm with a score of 2 and (8.04 ± 0.88) mm with a score of 1 against *P. acnes* and *S. aureus*, which was smaller than the (12.64 ± 1.19) mm with a score of 2 and (12.54 ± 1.15) mm with a score of 2 for TNCs. CMC-OHA/TNCs had a highly sensitive inhibition circle diameter of (23.72 ± 0.70) and (33.12 ± 1.40) mm against *P. acnes* and *S. aureus*, both with a score of 4. CMC -OHA/TNCs was more effective against *P. acnes* and *S. aureus* than TE crude suspension and TNCs, and its effect was comparable to erythromycin. In addition, CMC-OHA hydrogels also exhibited antibacterial effect, with an inhibition circle diameter of (15.60 ± 1.12) mm and score 3 against *P. acnes*, and an inhibition circle diameter of (17.60 ± 1.32) mm and score 3 against *S. aureus*. Overall, the incorporation of TNCs into CMC-OHA hydrogels could partially increase the bactericidal effect of TNCs against *P. acnes* and *S. aureus*. It was inferred that the superior antibacterial activity of CMC-OHA/TNCs might be due to the synergistic effect of TNCs with chitosan. The superior antibacterial activity of the hydrogels was attributed to the inherent properties of chitosan. The highly charged polycationic structure of chitosan generated intense electrostatic interaction with the negatively charged phospholipid membrane components of bacteria, leading to bacterial death through membrane damage and subsequent leakage of contents [31].

*P. acnes* and *S. aureus* played an important role in the pathogenesis of acne. *P. acnes* promoted the production of lipase and oxygen free radicals, affected the normal secretion of the sebaceous glands and activated the inflammatory response through intrinsic and adaptive immunity [32]. In addition, *P. acnes* promoted the formation of *S. aureus* biofilms and enhanced the virulence of *S. aureus* [33]. Due to the chronic nature of acne, topical medication played an important role, with antibiotics, retinoic acid and benzoyl peroxide being the main agents available [34]. Long-term use of antibiotics carried the risk of drug resistance; retinoic acid had many side effects and was teratogenic, and benzoyl peroxide was associated with skin irritation [35,36]. Therefore, there was an urgent need for a clinically effective drug that had an antibacterial effect without significant adverse effects. In recent years, there had been an increase in the number of reports of herbal remedies for acne, but most of them were mainly taken internally. A number of experiments had demonstrated the inhibitory effect of various herbal extracts on *Propionibacterium acnes* in vitro [9].

The results of one study showed that the MIC of clindamycin against *P. acnes* was 0.098 μg/mL and the MIC of erythromycin against *S. aureus* was 0.098 μg/mL, much lower than the MIC of CMC-OHA/TNCs against *P. acnes* and *S. aureus* in this study. This result showed that although CMC-OHA/TNCs had some bacterial inhibition, it was significantly weaker than the effect of antibiotics. Although the MIC of CMC-OHA/TNCs was higher than that of antibiotics, the inhibition circle results showed that *P. acnes* and *S. aureus* were equally susceptible to CMC-OHA/TNCs. In addition, there is currently no risk of resistance to CMC-OHA/TNCs compared to traditional antibiotics, and some findings determined that TE had good anti-inflammatory effects, so CMC-OHA/TNCs could control and improve the inflammatory response in the onset of acne while being antibacterial [37]. In summary, CMC-OHA/TNCs is a promising drug for the treatment of acne.

## 3. Materials and Methods

### 3.1. Materials

Tanshinone extract was obtained from (Xi’an Hongsheng Biotechnology Co., Ltd., Xi’an, China); Tanshinone IIA and cryptotanshinone control (≥98% HPLC)were obtained from (China Institute of Food and Drug Control, Beijing, China); Poloxamer 188 (P188) and Poloxamer 407 (P407) were obtained from (Beijing Fengli Jingqiu Trading Co., Ltd., Beijing, China); Sodium dodecyl sulfate (SDS), HA and CMC were obtained from (Tianjin Guangfu Fine Chemical Research Institute, Tianjin, China); Polyvinyl pyrrolidone K30 (PVP K30), hydroxypropyl methylcellulose E15 (HPMC-E15) and hydroxypropyl methylcellulose E30 (HPMC-E30) were obtained from (Shanghai Lanji Technology Development Co., Ltd., Shanghai, China); Chromatographic methanol was obtained from (Fisher Chemical Company, Hampton, NH, USA). Distilled water was produced by a laboratory ultrapure water machine. We also used standard strains of *P. acnes* and *S. aureus* and culture media (Xi’an Guolian Quality Testing Technology Co., Xi’an, China); 3T3-L1 cells (Pricella, Wuhan, China). Dulbecco’s modified Eagle-s medium (DMEM), fetal bovine serum (FBS), Trypsin- EDTA, and Penicillin-Streptomycin solution were purchased from Gibco (Waltham, MA, USA).

### 3.2. Preparation of TNCs

TNCs was prepared by the media milling technique with zirconium oxide beads as milling media. Briefly, TE drug (0.8%, *w*/*v*) was dispersed in 5.0 mL of distilled water containing stabilizer (0.2%, *w*/*v*), which was placed into the glass vial containing 4.0 mL of zirconia beads (with a diameter of 0.6–0.8 mm), and then milled under magnetic stirring (DF101S, Beijing Hengfeng Changwei Industrial and Trading Co., Ltd., Beijing, China) at a speed of 1400 rpm for 2 h to obtain TNCs.

### 3.3. Optimization of Prescriptions and Processes for TNCs

In order to compare the stabilizing abilities of different stabilizers, P188, P407, HPMC-E15, HPMC-E30, and PVP K30 were adopted for the preparation of TNCs, respectively. The effects of stabilizers and drug ratios on TNCs were also investigated. For further optimization of TNCs, a 3-factor, 3-level Box–Behnken design (BBD) was used to optimize the processes of TNCs. The second-order polynomial models and quadratic response surfaces were generated by Design Expert 13.0.5.0 (Minneapolis, MN, USA). The independent and dependent variables are listed in Table 4, along with their low, middle, and high levels.

### 3.4. Particle Size and Determination

The particle size and polydispersity index (PDI) of TNCs were determined by a Zeta-sizer (Malvern Nano-ZS Zetasizer, Worcestershire, UK). All samples were diluted with deionized water to avoid the multi-scattering phenomenon before measurement. Measurement of each sample was carried out at room temperature in triplicate and the results were recorded as an average.

### 3.5. Synthesis and Fourier Transform Infrared Spectroscopy (FTIR) Analysis of OHA

OHA was prepared by Sodium Periodate Oxidation [38]. Firstly, HA of 2.00 g was dissolved in 50 mL double distilled water and 1.30 g of NaIO4 was prepared in 5 mL aqueous solution and then added dropwise to HA solution. Next, the mixture was allowed to react at an ambient temperature in the dark for 12 h to ensure a complete reaction. Then, 0.5 mL of ethylene glycol was added to the reaction product with a rotating speed of 600 rpm for 1 h to quench unreacted NaIO4. Finally, the product was placed in a dialysis bag (MWCO 3500 kDa) and purified through exhaustive dialysis against double distilled water for three days followed by lyophilization to obtain OHA powder [39].

### 3.6. Preparation of CMC-OHA/TNCs

**Figure 12:**
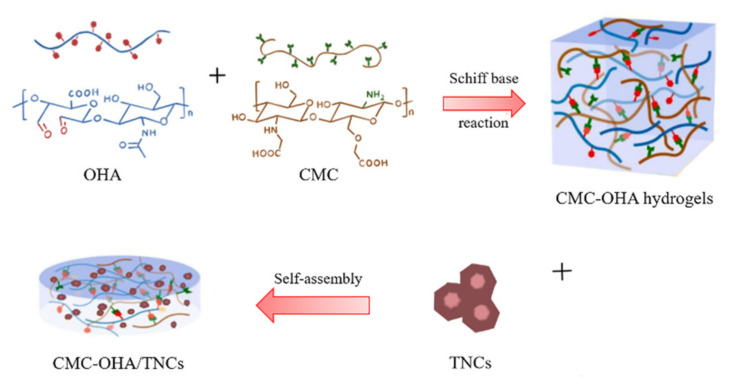
Schematic representation of the formation of CMC-OHA/TNCs.

OHA and CMC were dissolved in distilled water, respectively, to form solutions of different concentrations. Equal volume of OHA and CMC solutions were mixed well and kept in a water bath at 37 °C until gel formation. The tilting method was used to determine the gel formation time. After the OHA and CMC solution contacting with each other, the Celine bottle was tilted to observe the flow of the solution and the timing was stopped when the system stopped flowing when the bottle was tilted. To encapsulate TE into the hydrogels, the OHA powder was dissolved in the TNCs and subsequently mixed with the CMC solution in a water bath at 37 °C until solidification, resulting in CMC-OHA/TNCs. The schematic illustration of the formation of CMC-OHA/TNCs is shown in Figure 12.

### 3.7. Morphology of CMC-OHA/TNCs

The morphologies of TNCs and CMC-OHA/TNCs were analyzed by scanning electron microscopy (SEM) (S-4800, Hitachi Technologies Corporation, Tokyo, Japan). TNCs, CMC-OHA hydrogels and CMC-OHA/TNCs were applied to tin foil and left to dry at room temperature for 24 h; TE powder was used as a control. Samples were attached to the sample stage with conductive tape and a vacuum spray of the gold film was performed before observation with scanning electron microscopy (SEM). The SEM was performed at an excitation voltage of 15 kV.

### 3.8. X-ray Powder Diffraction (XRD) of CMC-OHA/TNCs

The crystalline nature of TE powder, TNCs freeze-dried powder, powdered physical mixture of hydrogels, and CMC-OHA/TNCs were examined by XRD, respectively. TNC was lyophilized by a freeze dryer (Lab-1A-50, Beijing Bo Yikang Experimental Instrument Co., Ltd., Beijing, China) to obtain a dry sample. The X-ray diffractograms were recorded by an X-ray diffractometer (D/Max-2500PC, Rigaku, Tokyo, Japan) with a Cu line as the source of radiation. The X-ray diffractograms were performed in a step scan mode with a current of 25 mA and a voltage of 40 kV over the angle range of 7 °C to 55 °C with 1 °C/min scan speed to estimate the crystallinity of the samples.

### 3.9. Determination of Cryptotanshinone and Tanshinone IIA

CMC-OHA/TNCs of 1.00 g was placed into 100 mL of methanol and sonicated for 10 min to cause the cryptotanshinone and tanshinone IIA to completely dissolve in methanol. The samples were filtered through a 0.2 μm membrane filter, and the content of cryptotanshinone and tanshinone IIA were determined by HPLC (LC-20A, Shimadzu, Tokyo, Japan). Analyses were performed on inertsil ODS-3 column (250 × 4.6 mm, 5 μm, Shimadzu, Tokyo, Japan) with column temperature maintained at 30 °C. The mobile phase consisted of methanol and 0.1% phosphoric acid (85:15, *v*/*v*). The flow rate was 1 mL·min^−1^ and the detection wavelength was 254 nm. Tests of each sample were carried out three times and the results were recorded as an average.

### 3.10. In Vitro Transdermal Permeation

The in vitro transdermal permeation of CMC-OHA/TNCs was examined using a Franz diffusion cell (RYJ-6B) [40]. The abdominal skin of male SD rats was taken, the subcutaneous tissue, blood vessels and fat were scraped off, rinsed repeatedly with saline and checked for integrity. The skin was fixed on the diffusion cell with the stratum corneum facing upwards. Phosphate buffer (pH = 5.0, 5.5, 7.4) was used as the receiving solution to bring the skin and receiving surface into complete contact [29]. The diffusion cell was kept at 37 °C and stirred continuously at 300 rpm. CMC-OHA/TNCs of 0.50 g was tightly applied to mouse skin. Samples (0.5 mL) were withdrawn at 0.5, 0.75, 1, 2, 4, 8, 12, 16, 24, 36, 48 and 72 h, and immediately supplemented with equal volume of the release medium at the same temperature. Samples were filtered through 0.22-μm membrane filters and determined by HPLC as described in Section 3.9.

### 3.11. CCK-8 Assay

3T3-L1 cells were cultured in DMEM supplemented with 10% (*v*/*v*) FBS and penicillin (100 U/mL)/streptomycin (100 μg/mL), and grown in an incubator at 37 °C supplemented with 5% CO_2_ under fully humidified conditions. CMC-OHA/TNCs were dissolved in complete growth medium to form media containing different concentrations of CMC-OHA/TNCs (0, 25, 50, 100, 200 and 400 μg/mL). 3T3-L1 cells were seeded in a 96-well plate at a density of 1 × 10^5^ cells/mL and incubated for 24 h. Then, the 3T3-L1 cells were treated with 0, 25, 50, 100, 200 and 400 µg/mL of CMC-OHA/TNCs for 48 h. Cell viability was evaluated with CCK-8 (Pricella, Wuhan, China) according to the manufacturer’s instructions. Absorbance was measured at 450 nm using a spectrophotometer (BioTek Instruments, Winooski, VT, USA), and cells cultured with complete growth medium were used as control.

### 3.12. In Vitro Antibacterial Activity Assay

Minimum inhibitory concentration (MIC) and minimum bactericidal concentration (MBC) were measured by doubling dilution according to the Clinical and Laboratory Standards Association guidelines to compare the antimicrobial activity of TE crude suspension, TNCs and CMC-OHA/TNCs. The TE crude powder was dispersed in distilled water containing P407 (0.2%, *w*/*v*) and then stirred with a magnetic stirrer at 600 rpm to obtain a suspension of TE crude powder. First, 100 µL of Luria Bertani (LB) medium was placed in each well of a sterile 96-well microtiter plate. Both TE crude suspension and TNCs were diluted to 512 µg/mL with LB medium, respectively. Dilutions of TE crude suspension, TNCs and CMC-OHA/TNCs were added to the set-up wells separately, followed by serial 2-fold dilutions. The final solutions were obtained at concentrations of 0.002, 0.004, 0.008, 0.016, 0.031, 0.063, 0.125, 0.250, 0.500, 1.000 mg/mL. Then, 100 µL of the prepared bacterial dispersion at a concentration of 10^5^ CFU/mL was pipetted into each well, except for the sterile control wells. Finally, the MIC value was evaluated visually by comparing the culture turbidity. Next, 5 µL of the medium was picked from the dilutions without significant bacterial growth to assess the MBC of the TE crude suspension, TNCs and CMC-OHA/TNCs being tested. Selected cultures were applied to a sterile LB agar medium and incubated at 37 °C for 24 h. Thereafter, bacterial colonies were counted. The MBC was determined to be the lowest concentration at which fewer than five colonies were detected after incubation on LB nutrient agar.

The inhibition zones of TE crude suspension, TNCs, CMC-OHA hydrogels, and CMC-OHA/TNCs against *P. acnes* and *S. aureus* were determined for comparing their bactericidal activities. A total of 100 µL of the bacterial suspension (10^8^ CFU/mL) was spread on LB nutrient agar to prepare a confluent ground for bacterial growth. Then, a 0.5 cm diameter circle was used to apply an equal mass of 0.20 g of the drug to each of the surfaces of the medium containing the bacteria. The diameter of the inhibition zones at each administration site was measured and scored after 48 h (Table 5).

### 3.13. Statistical Analysis

SPSS Statistics Version 22.0 (SPSS Inc., Chicago, IL, USA) was used to perform statistical analysis. An independent *t*-test was used to evaluate the significance of the difference between the two independent groups. The difference was accepted to be significant if *p* < 0.05.

## 4. Conclusions

TE is a promising drug for acne, although low bioavailability due to its poor water solubility limits its clinical application. Improving the solubility could increase the dermal bioavailability, and nanosizing is a useful way to improve solubility. In this study, TNCs were prepared using a media milling technique and the optimal prescription and process were obtained by single-factor experiments and a three-factor, three-level Box–Behnken statistical design. The OHA powder obtained from the oxidation reaction was dissolved in TNCs and self-assembled with CMC solution to form CMC-OHA/TNCs. In vitro characterization experiments demonstrated that nanosized TE reduced its crystallinity, and the TNCs could be encapsulated as nanocrystals in a hydrogel as well as the good pH responsiveness and biocompatibility of CMC-OHA/TNCs to release the drug in a responsive manner in the skin of acne lesions. Additionally, the preliminary pharmacodynamic exploration indicated that TNCs and CMC-OHA/TNCs had lower MIC and MBC than TE and that loading TNCs into CMC-OHA hydrogels increased the antibacterial activity of TNCs. However, further studies are needed to fully explore these formulations, such as extensive pharmacokinetic, histopathological and toxicity studies. In conclusion, nanocrystalline hydrogels could be an effective transdermal delivery system that could be applied in the future to improve the dermal delivery of drugs with poor aqueous solubility.

## Figures and Tables

**Table 1:** Experimental arrangement with response of Box–Behnken design.

Run	Independent Variables			Response Value	
	X_1_ (mL)	X_2_ (rpm)	X_3_ (h)	Y_1_ (nm)	Y_2_
1	3	1400	4	281.2	0.2328
2	4	900	6	335.5	0.2520
3	4	900	2	260.6	0.2148
4	4	1400	4	267.5	0.2220
5	5	900	4	220.8	0.2280
6	4	1900	6	297.9	0.2316
7	5	1400	6	262.8	0.2256
8	0	1900	2	325.2	0.2616
9	5	1400	2	230.8	0.2424
10	3	1400	6	338.0	0.2520
11	4	1400	4	264.8	0.2196
12	4	1400	4	266.1	0.2148
13	5	1900	4	249.6	0.2196
14	4	1400	4	257.9	0.2184
15	3	900	4	286.4	0.2160
16	3	1400	2	297.0	0.2280
17	4	1400	4	268.5	0.2148

**Table 2:** Summary of results of regression analysis for measured responses.

Variables	*p*-Value	
	Y_1_	Y_2_
Model	<0.0001	0.0001
X_1_	<0.0001	0.2440
X_2_	0.0488	0.0122
X_3_	0.0008	0.2079
X_1_X_2_	0.0581	0.0109
X_1_X_3_	0.5657	0.0009
X_2_X_3_	0.0003	<0.0001
X_1_^2^	0.0065	0.4034
X_2_^2^	0.0511	0.0372
X_3_^2^	<0.0001	<0.0001
R^2^	0.9776	0.9727

**Table 3:** MIC and MBC of TE crude suspension and TNCs against *P. acnes* and *S. aureus* determined using doubling dilution.

Formulation		Concentration (μg/mL)		
	*P. acnes*		*S. aureus*	
	MIC	MBC	MIC	MBC
TE crude suspension	125	250	125	64
TNCs	31	63	63	63
CMC-OHA/TNCs	16	63	31	31

**Table 4:** Variables used in Box–Behnken design.

	Level		
	Low (−1)	Medium (0)	High (+1)
Independent variables			
The amount of grinding media	3	4	5
Grinding media	900	1400	1900
Grinding media	2	4	6
Dependent variables	Constraints		
Y_1_ = Particle size(nm)	Minimize Minimize		
Y_2_ = PDI			

**Table 5:** Scoring table of diameter of inhibitory zones.

Diameter of Inhibitory Zones	Sensitivity	Score
Diameter > 20 mm	Extremely sensitive	4
20 mm > Diameter ≥ 15 mm	Highly sensitive	3
15 mm > Diameter ≥ 10 mm	Medium sensitivity	2
10 mm > Diameter ≥ 7 mm	Hypersensitive	1
7 mm > Diameter	Insensitive	0

## Data Availability

All data available are reported in the article.

## Abbreviations

CMC, carboxymethyl chitosan; OHA, oxidized hyaluronic acid; TNCs, tanshinone extract nanocrystals; CMC-OHA/TNCs, tanshinone extract nanocrystal hydrogels; MIC, minimum inhibitory concentration; MBC, minimum bactericidal concentration; DHT, dihydrotestosterone; HA, hyaluronic acid; XRD, X-ray diffraction; SEM, scanning electron microscopy; P188, Poloxamer 188; P407, Poloxamer 407; SDS, Sodium dodecyl sulfate; PVP K30, Polyvinyl pyrrolidone K30; HPMC-E15, hydroxypropyl methylcellulose E15; HPMC-E30, hydroxypropyl methylcellulose E30; PDI, polydispersity index; FTIR, Fourier transform infrared spectroscopy; BBD, Box–Behnken design; LB, Luria Bertani; TE, Tanshinone extract; DMEM, Dulbecco’s modified Eagle-s medium; FBS, fetal bovine serum; CCK-8, counting kit-8.
